# Chemopreventive Potential of Flavonoids in Oral Squamous Cell Carcinoma in Human Studies

**DOI:** 10.3390/nu5072564

**Published:** 2013-07-08

**Authors:** Marcello Iriti, Elena Maria Varoni

**Affiliations:** 1 Department of Agricultural and Environmental Sciences, Milan State University, via G. Celoria 2, Milan 20133, Italy; 2 Department of Mining and Materials Engineering, McGill University, University Street 3610, Montreal, QC H3A 2B2, Canada; E-Mail: elena.varoni@mail.mcgill.ca; 3 Department of Biomedical, Surgical and Dental Sciences, Milan State University, Milan 20133, Italy; 4 Department of Health Sciences, University of Eastern Piedmont “A. Avogadro”, Novara 28100, Italy

**Keywords:** oral cancer, polyphenols, anthocyanins, tea, black raspberry, nanochemoprevention, nanoparticles, transmucosal oral delivery

## Abstract

Evidence available from nutritional epidemiology has indicated an inverse association between regular consumption of fruits and vegetables and the risk of developing certain types of cancer. In turn, preclinical studies have attributed the health-promoting effects of plant foods to some groups of phytochemicals, by virtue of their many biological activities. In this survey, we briefly examine the chemopreventive potential of flavonoids and flavonoid-rich foods in human oral carcinogenesis. Despite the paucity of data from clinical trials and epidemiological studies, in comparison to *in vitro*/*in vivo* investigations, a high level of evidence has been reported for epigallocatechin gallate (EGCG) and anthocyanins. These flavonoids, abundant in green tea and black raspberries, respectively, represent promising chemopreventive agents in human oral cancer.

## 1. Introduction

Dietary habits rich in fruits, vegetables, legumes and whole cereals have been significantly correlated to a reduced risk of chronic degenerative disorders, particularly cardiovascular diseases and certain types of cancer [1,2]. Health-promoting effects of plant foods have been ascribed to their content in secondary metabolites. These phytochemicals, by virtue of their biological activities, possess different molecular and biochemical targets both in healthy and diseased cells. Antioxidant, anti-inflammatory, antimicrobial, pro/anti-apoptotic and vasodilating activities are only some of the properties of plant natural products responsible for their anticancer, cardio- and neuroprotective effects [3]. Plant tissues and, consequently, plant foods contain hundreds of bioactive secondary metabolites, including phenylpropanoids, isoprenoids and alkaloids [4]. Among these, phenylpropanoids have been particularly investigated in the two last decades, in order to ascertain health benefits attributed to diets rich in these metabolites. Only plants, including algae and some microorganisms, are able to synthesize phenylpropanoids from the free aromatic amino acids phenylalanine or tyrosine. The key enzyme in their biosynthesis is phenylalanine ammonia lyase, responsible for deamination of Phe, and, hence, in contrast to alkaloids, phenylpropanoids are ternary products, containing C, H and O. Hydroxybenzoates, hydroxycinnamates, coumarins, lignans, lignin and polyphenols are the main groups of phenylpropanoids. Therefore, the term polyphenols is not a synonymous of phenylpropanoids, but it refers to a vast group phenylalanine derivatives, in turn divided into flavonoids, stilbenes (including resveratrol) and proanthocyanidins (or condensed tannins) [5].

**Figure 1:**
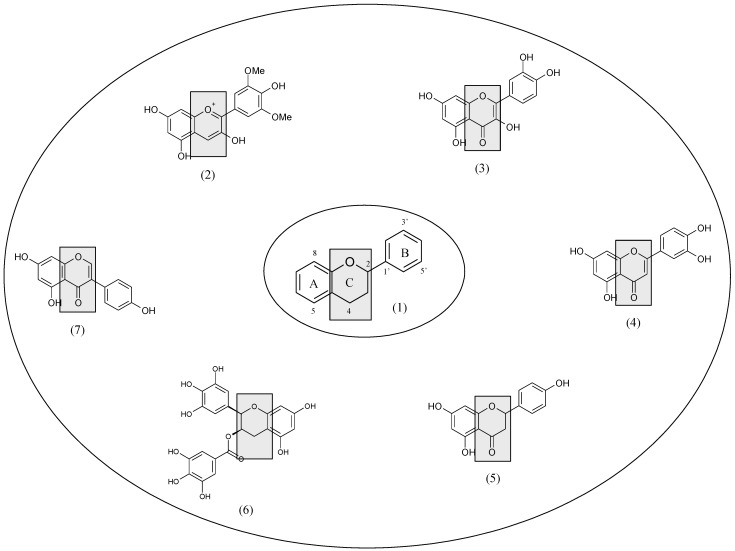
The flavan nucleus (**1**) is the basic structure of flavonoids, which include anthocyanidins (e.g., malvidin) (**2**), flavonols (e.g., quercetin) (**3**), flavones (e.g., luteolin) (**4**), flavanones (e.g., naringenin) (**5**), flavan-3-ols (e.g., epigallocatechin gallate) (**6**) and isoflavones (e.g., genistein) (**7**) differing in the level of oxidation and saturation of the C ring.

The flavonoid basic chemical structure is the flavan nucleus, a skeleton consisting of 15 carbon atoms arranged in three rings (C_6_–C_3_–C_6_): two aromatic rings (A and B) connected by a three-carbon-atom heterocyclic ring, an oxygen-containing pyran ring (C). The main classes of flavonoids (flavonols, flavanols, flavones, flavanones, isoflavones and anthocyanidins) differ in the level of oxidation and saturation of the C ring, while individual compounds within a class vary in the substitution pattern of the A and B rings (Figure 1).

Flavonols mainly include kaempferol, quercetin and myricetin aglycones (not bound to a carbohydrate moiety), whereas flavan-3-ols (or flavanols) provide catechin epimers [(+)-catechin and (−)-epicatechin], the monomeric units for proanthocyanidin biosynthesis. Flavanones are typical of citrus fruits (genus *Citrus*), mainly represented by aglycones hesperetin and naringenin, whereas apigenin and luteolin are widely diffused flavones. Isoflavones, a group of phytoestrogens which includes soy genistein and daidzein, are important constituents of the Fabaceae family, with the B ring in position 3 instead of 2. Anthocyanidins are the most abundant pigments in the plant tegumental tissues. Their conjugated derivatives, anthocyanins, mainly bound to sugars (glycones), hydroxycinnamates or organic acids, are water-soluble pigments conferring blue, dark blue, violet, red and purple hues to flowers, fruits and other plant organs. Anthocyanins are structurally based on six aglycones/anthocyanidins––malvidin, cyanidin, delphinidin, peonidin, pelargonidin and petunidin––which differentiate on the basis of number and position of hydroxyl groups and degree of methylation [6].

Oral cavity cancer is one of the most common and lethal head and neck malignancies. The overall incidence allows to consider these diseases the sixth more common form of cancer worldwide, with a high risk for recurrence (20%–30%) and a five-year survival rate of less than 50% [7,8]. Regional lymph node metastasis and loco-regional relapse are the main factors responsible for the poor survival rate of patients [7,8]. Even if head and neck cancers generally develop in the larynx and pharynx, they are intraoral in approximately 48% of cases, with a predominant location on the ventral/lateral lingual site or on the floor of the mouth. Less often, they can afflict others oral structures (gingiva and alveolar ridge, buccal mucosa, labial mucosa and hard palate). More than 90% of these cancers are classified histologically as oral squamous cell carcinoma (OSCC) [7,8].

Oral carcinogenesis is a highly complex, multifactorial process. The most important risk factors contributing to the etiology of oral cancer in Western countries are tobacco smoking and alcohol consumption [9]. Although drinking and smoking are independent risk factors, they have a synergistic effect, increasing the risk together [9]. Another relevant predisposing factor includes the presence of premalignant lesions associated to different degree of epithelial dysplasia [10]. Leukoplakia, proliferative verrucous leukoplakia, erythroplakia, erythroleukoplakia, oral submucous fibrosis and oral lichen planus display, to different extents, a certain propensity for tissutal transformation to OSCC. Moreover, viral infections and genetics may further contribute to carcinogenesis [10]. In particular, human papillomavirus (HPV) infection, mainly related to type 16, has been recently suggested to play a role in oral cancer development [11,12,13]. Finally, individuals that carry the fast-metabolizing alcohol dehydrogenase type 3 (ADH3) allele may be particularly vulnerable to the effect of chronic alcohol consumption, and they could be at increased risk to develop oral cancer [9,10].

## 2. Oral Cancer Chemoprevention and Preclinical Evidence

The transformation of a normal cell to cancer cell occurs through three distinct phases: initiation, promotion and progression. Initiation of cancer is due to the exposure of normal cells to carcinogenic and mutagenic agents. Initiated cells are irreversibly altered, and they are at higher risk of neoplastic transformation. However, initiation alone is not sufficient for tumorigenesis. In the promotion phase, cancer promoters convert the initiated cells into preneoplastic cells. Progression involves a stepwise evolution of preneoplastic cells into neoplastic cells with a higher degree of malignancy. Then, the tumoral mass acquires an aggressive characteristic, such as invasion and metastasis.

In general, chemoprevention consists of the use of chemicals for reversal, suppression or prevention of the transformation of premalignant cells to a malignant geno/phenotype. Therefore, chemopreventive agents are classified into two categories:

1. blocking agents prevent carcinogens from reaching and reacting with critical cell target sites, *i.e.*, they prevent metabolic activation of carcinogens and stimulate their detoxification [14];

2. suppressing agents may impair the neoplastic transformation in target cells, at both early and late stages of carcinogenesis [14].

So far, the potential of flavonoids on oral health and as cancer chemopreventive agents has been demonstrated in many preclinical studies [15,16]. Indeed, with regard to oral cancer, many flavonoids have been investigated *in vitro* and *in vivo*. Two isoflavones, genistein and biochanin A, decreased the cell growth of OSCC cell lines with an IC_50_ of 50 µM and inhibited phosphorylation of ERK (extracellular signal-regulated kinase) and Akt, a mitogen-activated protein kinase (MAPK) and a serine/threonine protein kinase, respectively, involved in oral cancer proliferation [17]. The flavone baicalein induced G1 phase arrest in oral cancer cells by enhancing the degradation of CDK4 (cyclin-dependent kinase) and cyclin D1, and activating AhR (aryl hydrocarbon receptor) [18]. Similarly, quercetin (a flavonol) inhibited OSCC cell proliferation via both G1 phase arrest and mitochondria-mediated apoptosis, besides decreasing cell migration and invasion [19]. A p53-dependent apoptotic pathway induced by vitexin (a flavone) was also demonstrated in oral cancer cell [20]. In 7,12-dimethylbenz(a)anthracene (DMBA)-induced experimental oral carcinogenesis in Syrian hamsters, administration of the flavone apigenin prevented the formation of oral tumors, by reducing oxidative stress and modulating the phase I and phase II detoxification cascade responsible for xenobiotic biotransformation [21,22].

However, EGCG and other flavanols from *Camellia sinensis* L. are the most investigated flavonoids in oral cancer chemoprevention. Recently, excellent reviews have reported the cytotoxic activity of these compounds on different OSCC cell lines and in both chemical-induced and xenograft models of OSCC in rodents, also investigating the molecular mechanisms involved in chemoprevention [23,24,25]. In particular, *in vitro* effects of EGCG on oral cancer cells included three main steps: (i) inhibition of cell proliferation via apoptosis induction and cell cycle arrest; (ii) modulation of transcription factors, namely nuclear factor-κB (NF-κB) and activator protein (AP)-1; (iii) and reduction of cell migration and invasion by decreasing the production of matrix metalloproteinases. In different animal models of oral carcinogensis, tea polyphenols reduced oxidative stress and phase I enzymes while inducing phase II enzyme activities [23,24]. On the basis of preclinical evidence, clinical trials have been recently conducted, thus, in the next section, we will briefly deal with human studies on the chemopreventive potential of flavonoids, mainly focusing of EGCG and green tea.

## 3. Epidemiological Studies

One of the first studies correlating increased oral cancer risk to low intake of fruits and vegetables––rich in flavonoids––was published three decades ago. Increased consumption of fruits and vegetables was shown to be protective against oral cancer, when statistically controlled for demographic traits, tobacco and alcohol use, relative weight and intake of other food items [26]. More recently, high fruit and vegetable intake was associate with reduced risk of head and neck cancer, in a pooled analysis by the INHANCE (International Head and Neck Cancer Epidemiology) consortium which collected data from 22 case-control studies with 14,520 cases and 22,737 controls. Interestingly, red and processed meat intake was positively associated with increased risk of head and neck cancer [27].

The consumption of flavonoids has been inversely correlated to the risk of oral cancer in two case-control studies conducted in Uruguay and Italy, which reported relative risks (RRs) for the highest level of flavonoid intake of 0.8 and 0.56, respectively [28,29]. In particular, in the Italian study, a significant inverse association was found for flavanones, flavonols and total flavonoids, with odds ratios (ORs) for the highest *versus* the lowest quintile of intake of 0.51, 0.62 and 0.56, respectively. No significant association emerged for other classes of flavonoids, *i.e.*, isoflavones (OR, 0.90), anthocyanidins (OR, 0.86), flavan-3-ols (OR, 0.84) and flavones (OR, 0.75) [29].

The association between green tea consumption and oral cancer risk was studied in the Japanese Collaborative Cohort Study. A total of 20,550 men and 29,671 women aged 40–79 years without any history of oral cancer were included at baseline in this prospective study. During a mean follow-up period of 10.3 years, 37 oral cancer cases were identified. For women, the hazard ratios (HRs) of oral cancer for a green tea consumption of 1–2, 3–4 or 5 cups/day were 0.51, 0.60 and 0.31, respectively, compared to 1 cup/day. For men, the inverse association was slightly lower, and, in any case, it did not reach statistical significance due to the relatively low number of cancer cases included in the analysis [30].

Tea intake was not associated with oral cancer in the Cancer Prevention Study II, a large prospective US cohort study that began in 1982, involving 968,432 men and women, cancer-free at enrolment [31]. However, in this study, the type of tea consumed––black or green—was not specified. Whereas catechins are found in green tea, the main constituents of black tea are theaflavins and thearubigins, which are formed by the oxidation and polimerization of catechins during fermentation and, presumably, possess different biological activities [32].

Conversely, in the French ICARE (Investigation of Occupational and Environmental Causes of Respiratory Cancers) study, a large multicenter, population-based, case-control study on lung and upper aerodigestive tract cancers was carried out from 2001 to 2007 in 10 French administrative areas, an inverse association between oral cavity cancer and tea and/or coffee intake was observed. For the highest quartile of exclusive tea or coffee consumption, ORs were 0.39 and 0.60, respectively, with a synergistic effect when both consumed by the same subject. No difference in risk between men and women was reported [33].

Finally, according to a meta-analysis by the Cochrane Collaboration of 51 studies including more than 1.6 million participants, evidence that the consumption of green tea may reduce the risk of cancer is conflicting. Drinking green tea remains unproven in oral cancer prevention, but appears to be safe for moderate, regular, and habitual use [34].

## 4. Clinical Studies

To the best of our knowledge, tea catechins are the only flavonoids investigated in clinical studies on oral cancer. The first clinical trial using green tea for treatment of an oral premalignant lesion was a double-blind, placebo-controlled, randomized, phase II clinical trial in 59 patients with oral mucosa leukoplakia receiving either 3 g/day of a mixed tea product in oral capsules, in four divided doses, plus 10% mixed tea ointment in glycerine topically, or placebo plus topical glycerine. Applying the tea extract directly to the lesions may improve the local concentrations of the active constituents. The mixed tea was composed of a dried mixture of water-soluble green tea extract, green tea polyphenols and tea pigments (theaflavins and thearubigins). After six months of intervention, the size of oral lesions decreased in 37.9% and increased in 3.4% of the 29 tea-treated patients, whereas they decreased in 10.0% and increased in 6.7% of the 30 subjects in the placebo group [35].

Administration of a green tea extract (2000–2500 mg/day) to smokers for four weeks reduced DNA damage in oral keratinocytes. Moreover, cell growth was also inhibited, the percentage of cells in S phase decreased, cells accumulated in G_1_ phase, DNA content became more diploid and less aneuploid, and apoptotic markers were upregulated [36].

Another randomized, placebo-controlled, phase II trial evaluated the chemopreventive potential of green tea extract on oral cancer. The effects of administration of green tea capsules containing 13.2% of EGCG (26.9% of catechins) were examined on 41 patients with one or more histologically confirmed, bidimensionally measurable oral premalignant lesions at high risk of malignant transformation. Patients were randomized to receive green tea extract at 1.0 g/m^2^ (*n* = 10), 0.75 g/m^2^ (*n* = 9), 0.5 g/m^2^ (*n* = 11) or placebo, three times daily for 12 weeks. The efficacy was determined by the disappearance of all lesions (a complete response) or 50% or higher decrease in the sum of diameters of all measured lesions (a partial response). The authors found that the two high-dose arms (0.75 and 1.0 g/m^2^) revealed higher clinical response rates (58.8%) than 0.5 g/m^2^ (36.4%) or placebo (18.2%), even if rates did not reach statistical significance, suggesting a dose-response effect of green tea extract. The latter was well tolerated, with only insomnia (with higher doses, possibly due to the caffeine present in the extract), diarrhea and oral/neck pain. At a median follow-up of 27.5 months, there was no difference in oral cancer-free survival between the green tea extract arm and the placebo arm [37]. Finally, in patients with oral field cancerization, at a high risk for developing recurrent oral precancerous and cancer lesions, application of EGCG in a form of mouthwash for seven days decreased the expression levels of some biomarkers of oral carcinogenesis, though not statistically significant. It is noteworthy that detectable levels of EGCG were measured in saliva, but not in plasma, thus demonstrating local bioavailability of this catechin in oral mucosa without significant systemic absorption [38].

## 5. Oral Bioavailability of Flavonoids

Studying the biological activity of phytochemicals in humans, the knowledge of their pharmacokinetic and bioavailability is crucial. For food bioactive components, oral bioavailability is defined as the fraction of substances, obtained from ingested foods, that reach systemic circulation for further delivery to target tissues and organs, where they subsequently exert their biological activities. Flavonoids are recognized as xenobiotics by the human body. Consequently, to produce beneficial effects after ingestion, they have to be absorbed and metabolized before being delivered to target tissues and organs by the bloodstream [32,39]. Though biological activities of flavonoids have been demonstrated in many preclinical models, the effective concentrations *in vitro* (sub- to low-micromolar levels) are at least one order of magnitude higher than those normally measured in human plasma (tens to hundreds of nanomolar) [40]. To amount to effective concentrations at their sites of action, ingested flavonoids have to overcome a number of barriers represented by the complex structures of the gastrointestinal tract [41]. In general, the bioavailability of dietary flavonoids is not only limited by their physicochemical properties, but also because of active efflux by multidrug resistance-associated proteins or extensive biotransformation by phase I and phase II enzymes, including the first-pass hepatic metabolism, and gut microbiota [32]. In particular, the dietary forms of flavonoids, flavonoid glycosides, are considered inactive; they thus have to first be hydrolyzed to their active aglycones at enteric level. Significantly, hydrolysis of quercetin and genistein glucosides to their aglycones has been reported in human oral cavity, *i.e.*, in saliva and epithelial cells, due to both bacterial and epithelial β-glucosidases. Interestingly, hydrolysis was limited to glucose conjugates: other glycosides either were hydrolyzed very slow or were resistant to salivary hydrolysis. A remarkable inter-individual variability in hydrolysis rate was thus observed. Cytotoxicity of quercetin and genistein was also demonstrated *in vitro* on oral cancer cells, thereby suggesting that the aglycones formed in the oral cavity may exert anticancer activity [42]. More recently, intraoral bioactivation of anthocyanins via microbial, salivary and epithelial β-glucosidases has been described, with a high inter-individual variability. The authors also reported that, comparable to small intestine, the hydrolytic, phase II and efflux transporting enzymes necessary for local enteric recycling are present and functional in human oral mucosa [43]. Glucuronidated phase II anthocyanin conjugates were also detected in saliva [43].

## 6. Future Perspectives: Nanoparticles and Oral Transmucosal Delivery to Improve Systemic and Local Bioavailability

Poor bioavailability is the major drawback associated with the failure of many natural chemopreventive agents in clinical settings. In fact, despite the promising data obtained by chemoprevention by bioactive plant food components in preclinical studies, fallout of these results to humans showed limited success, possibly because of inefficient systemic delivery and low bioavailability of these promising chemopreventive agents. To date, a novel approach based on the use of nanotechnology to improve the outcome of cancer chemoprevention was developed, and a new concept termed “nanochemoprevention” was introduced, referring to encapsulation of chemopreventive agents in biocompatible nanoparticles [44,45].

Encapsulated EGCG in polylactic acid-polyethylene glycol nanoparticles retained its biological activity with over 10-fold dose advantage. *In vitro*, nano-EGCG was more cytotoxic to human prostate carcinoma PC3 cell lines, resulting in enhanced cellular apoptosis and inhibiting angiogenesis than non-encapsulated EGCG. A higher anticancer activity of the nanoformulation was also demonstrated in xenografted athymic nude mice [46].

Chemopreventive efficacy of naringenin-loaded nanoparticles was described in DMBA-induced experimental oral carcinogenesis in Syrian hamsters. Oral administration of naringenin-loaded nanoparticles completely prevented tumor formation as compared to free naringenin, and significantly reduced the degree of histological lesions. Additionally, in the buccal mucosa of DMBA-exposed animals, nanoparticles downregulated the expression of PCNA (proliferating cell nuclear antigen) and p53, and exerted higher anti-lipid peroxidative and antioxidant activities, when compared with free naringenin [47].

To benefit from flavonoid healthy effects, the local exposition of oral cavity to these compounds appears to be pivotal. In these terms, buccal and sublingual drug delivery technologies represent another promising and challenging approach. The transmucosal oral route is a method of systemic drug delivery that offers several advantages over gastro-enteric and parenteral routes. Oral mucosa is composed of three portions, from the top to the bottom: (a) an outermost layer of pluri-stratified squamous epithelium, with different degrees of cheratinization according to the oral site considered, for instance hard palate mucosa or gingival mucosa are more cheratinized than buccal mucosa; the basal epithelial layer is placed on (b) a basement membrane, consisting of collagen, laminin and fibronectin, in turn directly lying on (c) lamina propria or submucosa, as the innermost layer, comprised of connective tissue. Therefore, it represents a highly permeable and vascularized tissue which provides a rapid drug transport to systemic circulation, avoiding degradation by first-pass hepatic metabolism and pre-systemic elimination within the gastrointestinal tract. Furthermore, in comparison to intravenous and intramuscular administrations, transmucosal oral drug absorption is non-invasive, more comfortable and acceptable by patients. Buccal and sublingual delivery systems use mouthwashes, aerosol sprays, chewing gums, bioadhesive tablets, gels and patches to locally treat conditions such as gingivitis, oral candidosis, oral lesions, dental caries and xerostomia [48,49]. Although many drugs have been formulated for transmucosal oral delivery in order to treat both local and systemic conditions, only very few studies have been carried out on flavonoid and oral cancer [50,51].

Topical application to a selected mucosal site (posterior mandibular gingival mucosa) of a bioadhesive gel rich in anthocianins, containing 10% (w/w) black raspberry, produced detectable salivary and tissutal levels of black raspberry anthocyanins in healthy volunteers, and confirmed that gel-delivered flavonoids are readily released in the salivary environment and easily penetrate oral mucosa [52]. Similarly, in healthy volunteers, intraoral application of the same gel raised the levels of anthocyanins not only in saliva and oral tissues, but also in plasma samples [53]. Black raspberry gel was topically applied four times daily for six weeks to 17 patients with oral intraepithelial neoplasia, and pre- and post-treatment biopsies were evaluated. Seven patients showed histopathological improvement, six exhibited stable disease, and four evidence of progression. Then, the authors investigated the association between loss of heterozygosity and histological tissue modification, since loss of heterozygosity is associated with the development of many human cancers, including OSCC. A reduction in loss of heterozygosity at tumor suppressor gene-associated loci was observed: the study, thus reported a weak association between reduction in loss of heterozygosity and improvement in histopathology grade [54]. Finally, the same authors demonstrated that, in patients with oral premalignant lesions, topical berry gel application modulated the expression profile of oral intraepithelial neoplastic genes, suppressing those involved in RNA processing, growth factor recycling and inhibition of apoptosis. Treatment also reduced epithelial COX (cyclooxygenase)-2 protein, vascular density in the superficial connective tissues and genes associated with keratinocyte differentiation [55].

## 7. Conclusions

At the end of this brief survey, we can summarize that the current evidence of human oral cancer chemoprevention by flavonoids is still fragmentary and inconclusive, yet nonetheless promising, as suggested by both epidemiological and preclinical studies. It is noteworthy that the anticancer activity of these phytochemicals has been extensively reported.

Promising results have been recorded for EGCG and anthocyanins from green tea and black raspberries, respectively, though still inconsistent. Further studies, conducted following an evidence-based approach, are needed. In particular, phase III clinical trials with a high number of patients are required to confirm the efficacy of flavonoids on oral cancer prevention, as well as studies able to clarify the mechanisms involved in chemoprevention. Significantly, emerging technologies may maximize the chemopreventive potential of flavonoids, such as functionalization with nanoparticles and oro-transmucosal delivery, enhancing the efficacy of these phytoconstituents and improving their local and systemic bioavailability, the foremost weak point of nutritional therapy.

In perspective, the major outcome of these studies will be to increasingly figure out the relation of causality between phytochemicals and oral cancer prevention and/or treatment, thereby minimizing casuality. Indeed, benefits may be further enhanced by other “health-promoting” confounding factors, such as physical activity and other lifestyles associated to wellbeing conditions.

Finally, we have to take into account that, depending on dietary habits, we are exposed daily to hundreds of bioactive phytochemicals present in whole foods. Therefore, the additive and synergistic effects of all components, identifiable in fruits and vegetables, are more realistically responsible for the health-promoting properties ascribed to these foods, even if *in vitro*/*in vivo* studies mainly focused on the specific biological activities of only a few plant metabolites.
